# Fast Vessel Detection in Gaofen-3 SAR Images with Ultrafine Strip-Map Mode

**DOI:** 10.3390/s17071578

**Published:** 2017-07-05

**Authors:** Zongxu Pan, Lei Liu, Xiaolan Qiu, Bin Lei

**Affiliations:** 1 Key Laboratory of Technology in Geo-Spatial Information Processing and Application System, Chinese Academy of Sciences, Beijing 100190, China; lliu1@mail.ie.ac.cn (L.L.); xlqiu@mail.ie.ac.cn (X.Q.); leibin@mail.ie.ac.cn (B.L.); 2 Institute of Electronics, Chinese Academy of Sciences, Beijing 100190, China

**Keywords:** vessel detection, iterative CFAR approach, mean-shift based coarse detection, false alarms elimination, Gaofen-3 SAR images, ultrafine strip-map mode

## Abstract

This study aims to detect vessels with lengths ranging from about 70 to 300 m, in Gaofen-3 (GF-3) SAR images with ultrafine strip-map (UFS) mode as fast as possible. Based on the analysis of the characteristics of vessels in GF-3 SAR imagery, an effective vessel detection method is proposed in this paper. Firstly, the iterative constant false alarm rate (CFAR) method is employed to detect the potential ship pixels. Secondly, the mean-shift operation is applied on each potential ship pixel to identify the candidate target region. During the mean-shift process, we maintain a selection matrix recording which pixels can be taken, and these pixels are called as the valid points of the candidate target. The $l_{1}$ norm regression is used to extract the principal axis and detect the valid points. Finally, two kinds of false alarms, the bright line and the azimuth ambiguity, are removed by comparing the valid area of the candidate target with a pre-defined value and computing the displacement between the true target and the corresponding replicas respectively. Experimental results on three GF-3 SAR images with UFS mode demonstrate the effectiveness and efficiency of the proposed method.

## 1. Introduction

As an effective Earth observation means, synthetic aperture radar (SAR) technology has developed rapidly in recent years. SAR vessel detection is an important application in the field of marine surveillance and has received much attention recently. A SAR ship detection system usually consists of four stages: land masking, preprocessing, prescreening, and discrimination [1]. The land masking stage aims to distinguish the land from the sea surface. The preprocessing step aims to make the subsequent detection easier, and speckle filtering is a widely used preprocessing step. In the prescreening stage, certain algorithms are applied to search the entire image for potential ship pixels. Experience has shown that no algorithm can perfectly detect all ship pixels without introducing false alarms, thus a follow up discrimination stage is usually needed to remove these false alarms.

The land mask can be obtained by registering the image with an existing geographic map [1] or applying the edge detection method [2] or using a digital elevation model data to exclude the pixels with non-zero height [3]. The speckle filters are recommended as a preprocessing step by Ferrara et al. in their ship detection work [4,5]. Prescreening, also known as the detection step, is the core of the ship detection algorithm, and constant false alarm rate (CFAR)-based methods are the most widely used approaches for the detection phase. In the CFAR-based algorithms, a suitable statistical distribution is chosen to model the sea clutter, and then an adaptive threshold for a sliding reference window is computed given a constant false alarm rate. There are many CFAR detectors, such as the cell-averaging CFAR (CA-CFAR) and the two parameter CFAR (TP-CFAR) [1], the smallest-of CFAR (SO-CFAR) [6], the variability index CFAR (VI-CFAR) [7], the ordered-statistic CFAR (OS-CFAR) [8], and the trimmed-mean CFAR (TM-CFAR) [9]. The selection of the distribution model for the sea clutter is an important issue for the CFAR-based approach. For the SAR image with coarse resolution, the Gaussian distribution is always employed [10]. However, as the SAR image resolution increases, the Gaussian distribution is not suitable. The Weibull distribution and the K-distribution have been found to be useful in modeling the sea clutter over heterogeneous regions in SAR images with fine resolution [11]. In [12], Qin et al. applied a generalized gamma distribution-based CFAR detector, which achieves similar detection performance with much less running time compared with the K-distributed algorithm. An approximate estimator for the generalized gamma distribution parameters is derived in [13] to accomplish fast ship detection. Tao et al. proposed a truncated statistics (TS)-based CFAR detector [14], in which a fixed truncation ratio is used to exclude the potential ship pixels before estimating the parameter of the sea clutter distribution, and the exponential and gamma distributed sea clutter background models are taken into account. In [15], they further compared the gamma distribution, the K-distribution and a mixture of gamma distributions as the hypothesized sea clutter model with the TS-CFAR operator, and proposed a TS and segmentation-based CFAR detection (TS-SEG-CFAR) method, in which the expectation maximization (EM) algorithm is applied for better representing the distribution of the sea clutter. Basic CFAR detectors, like CA-CFAR and TP-CFAR, have well performance when there is a single target among locally homogeneous clutter. However, when there are multiple targets, the background area may include other targets, thus deteriorating the estimation of the sea clutter. An effective way to address this issue is to censor out the potential target pixels in the reference window before estimating the sea clutter distribution. Gao et al. proposed an automatic censoring algorithm for SAR target detection in [16], however the algorithm requires some prior knowledge about the size or the number of targets, which is hard to obtain in a real application. An iterative censoring scheme (ICS) has been proposed for SAR target detection [17]. The ICS method censors out the potential target pixels gradually by iteratively updating the target map, and is free from the need to obtain any prior knowledge. An et al. improved ICS in two aspects and proposed an improved ICS (IICS) method in [18]. One improvement is that not only the potential ship pixels but also their four-connected neighborhood pixels are censored out to increase the estimation accuracy of the sea clutter distribution, and the other is that a novel initial detection approach is applied to speed up the convergence of the ICS approach. Another kind of detection approach distinguishes the target from the sea clutter via feature extraction. A notch filter is applied to the polarimetric SAR image in [19] to generate the features and the candidate targets that have a different polarimetric feature from the sea are identified as the detected target. The variance weighted information entropy is used to extract the feature, which indicates the dissimilarity between the target and its neighborhood pixels [20]. Wang et al. fused the intensity and the spatial features together in a transform domain to boost the detected performance [21]. A saliency feature is obtained by the random forest-based hierarchical sparse model to generate candidate target regions in [22]. The superpixel-based method firstly applies a certain superpixel segmentation algorithm, such as the simple linear iterative clustering (SLIC) method [23], to generate several superpixels, and then the feature of each superpixel is computed as the criteria for the detection. The information theory is applied in [24] to extract the features of each superpixel, and the CFAR operator is employed after the SLIC superpixel segmentation in the SLIC-CFAR method [25] to detect the target.

The discrimination stage aims to reject false alarms, so that after this stage only ship targets are highlighted. False alarms can arise from SAR image artifacts, including side-lobes and azimuth ambiguities from land-based sources or strong point targets over the sea [26]. The presence of the azimuth ambiguities is well known, which are mainly caused by a too low pulse repetition frequency (PRF), under that circumstance there are significant returns from targets that have the same Doppler history as the signal (due to aliasing), and these returns are referred to as azimuth ambiguities [27,28]. Azimuth ambiguities are also called as replicas or ghosts, and the azimuth displacements between the true target and its replicas can be evaluated using the radar system parameters [29], therefore these azimuth ambiguities can be removed by searching for them in the image [3,26,30]. Vespe et al. analyzed in detail in which cases this searching-based method does not work well in [31]. Another effective technique removes azimuth ambiguities by designing certain filters, such as the Weiner selective filter [29]. Some recent CFAR-based approaches detect targets and remove false alarms simultaneously, such as the bilateral CFAR algorithm in [32], which combines the intensity and the spatial distributions to reduce the influence of SAR ambiguities. The features of the candidate target region are also used for the false alarm elimination, such as the contour feature in [22] and the Haar-like feature in [33].

Several methodologies detect ships and remove false alarms based on physical principles instead of standard image processing. Polarimetry and sub-aperture analyses are the most widely tools among these methodologies. Compared with the single polarimetric SAR image, the dual or the full polarimetric images contain the polarimetric information of ground objects, which can be exploited for target detection. The polarimetric information is exploited in [19] for forming the features that can distinguish ships from the sea clutter. By making use of the different symmetry properties of the sea clutter and the man-made targets in polarimetric SAR images, man-made targets at sea, such as ships, can be effectively detected [34]. The sub-aperture image, also known as sub-look image, can be formed by using just a portion of the full system bandwidth, at the cost of lower resolution than that of the original image [35]. The sub-aperture cross-correlation magnitude can improve the contrast between the targets and the sea clutter, consequently some approaches filter out the sub-aperture images as a preprocessing step for ship detection [36,37].

The Chinese Gaofen-3 (GF-3) satellite which can work in 12 different modes and is mainly used in the fields of ocean surveillance, disaster reduction, water conservancy and so on was launched in 2016 [38,39]. This paper aims at detecting vessels in Gaofen-3 (GF-3) SAR images with ultrafine strip-map (UFS) mode as fast as possible. UFS mode is one of the GF-3 SAR working modes, and when the satellite works under this mode, the signal is transmitted once with the horizontal or the vertical polarization and received twice with the horizontal and the vertical polarizations, thus the received signal has two channels corresponding to the horizontal and the vertical polarizations, respectively. Since ships have obvious differences in size, we focus on the detection of large vessels with lengths ranging from about 70 to 300 m. The proposed method consists of three parts. First, an iterative CFAR-based approach is applied to acquire the potential ship pixels. Then, a mean-shift based method is employed to identify the candidate target region, in which the $l_{1}$ norm regression [40] is used to compute the principal axis of the candidate target and then which pixels in the region belong to the candidate target can be obtained. Finally, false alarms are removed by calculating the valid area of each candidate target and applying the traditional azimuth ambiguities elimination approach. The remainder of this paper is organized as follows: Section 2 details the proposed method. In Section 3, experiments on three GF-3 SAR images with UFS mode are conducted to validate the effectiveness and efficiency of the proposed method, and the comparison with three state-of-the-art methods as well as the discussion is also presented in this section. Section 4 concludes this paper.

## 2. Proposed Method

### 2.1. Overall Scheme Based on Vessel Characteristic Analysis

**Figure 1:**
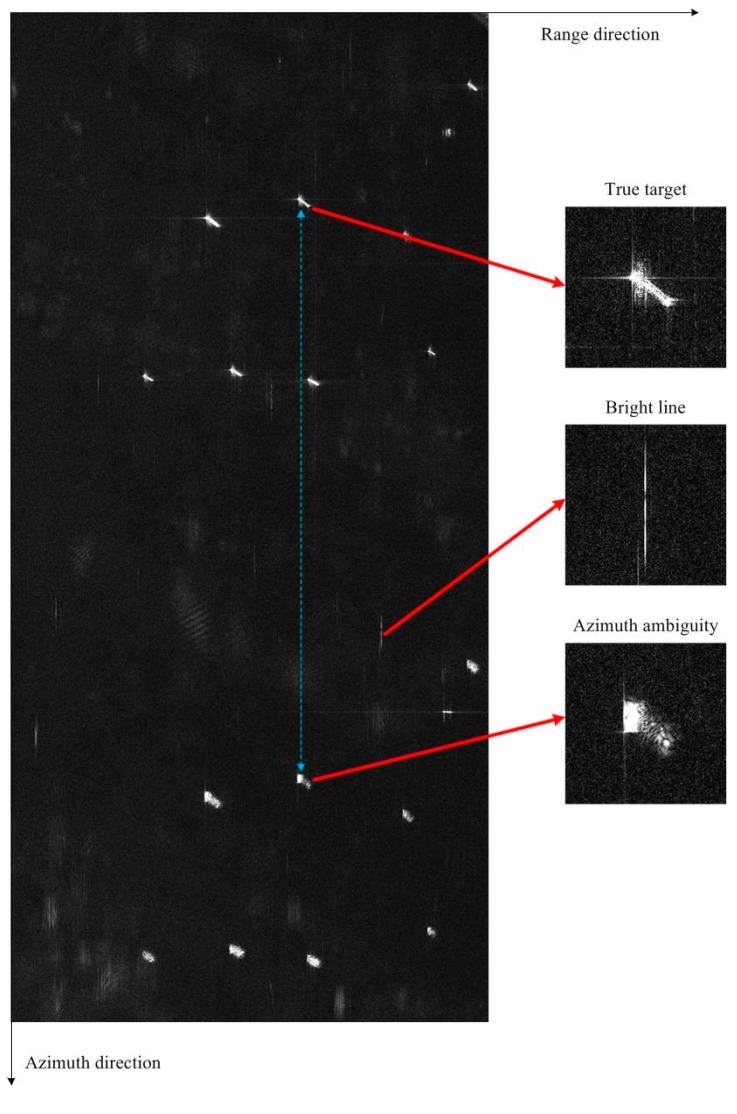
A GF-3 SAR image with vessels and two kinds of potential false alarms.

**Figure 2:**
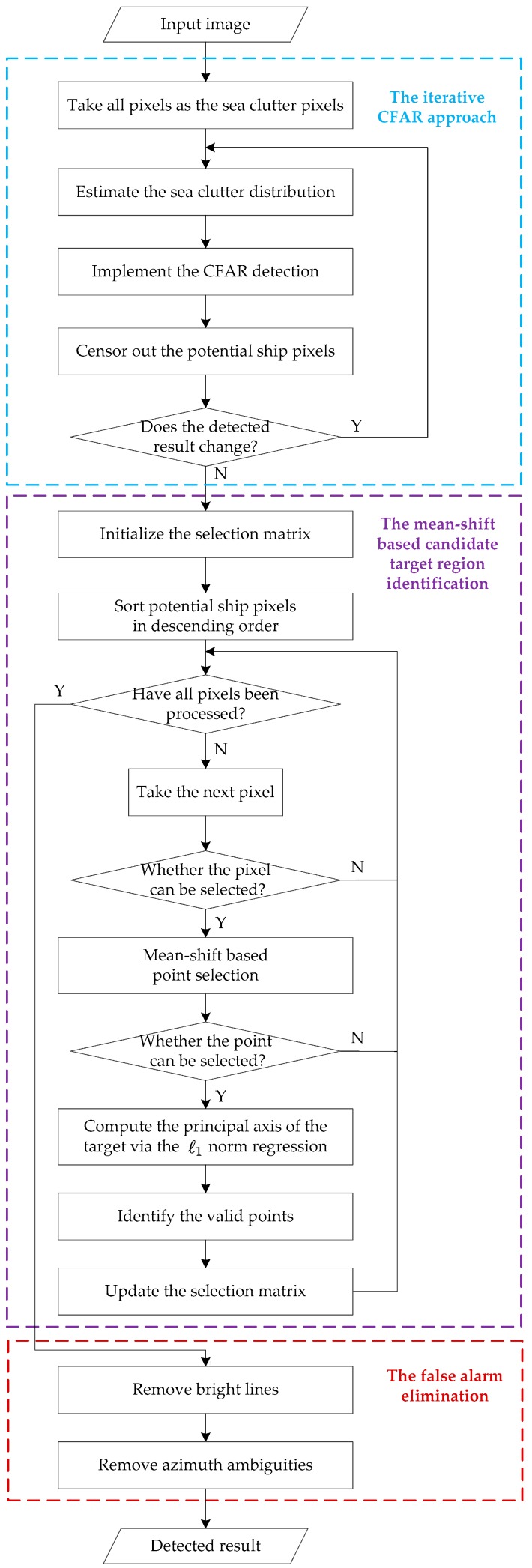
Workflow of the proposed method.

The backscattering characteristic of the vessel relies on many factors, including the material and structure of the vessel, the incidence angle, as well as other SAR parameters. Corns, edges, and surfaces give rise to even and odd bounces, which are the main scattering mechanisms. Superstructures of the vessel, such as the deck and the engine room will form dihedral or trihedral reflections, so they generate strong points in the image. Some studies put an effort on modeling the radar cross section (RCS) [41]. Figure 1 shows a GF-3 SAR image with several vessels, whose acquisition parameters are listed in Table 1 (Image #1). It can be seen from Figure 1 that the vessel is evidently brighter than the sea clutter, which is the basis for vessel detection. The proposed method consists of three steps: the iterative CFAR approach, the mean-shift-based candidate target region identification, and the false alarm elimination, which will be detailed in the following three sub-sections, and the workflow of the method is presented in Figure 2.

Step 1, the iterative CFAR approach. The iterative CFAR approach is a censoring scheme based method, in which the potential ship pixels are censored out iteratively to improve the estimation accuracy of the sea clutter distribution.

Step 2, the mean-shift based candidate target region identification. A mean-shift based method is used to identify the candidate target region, and the $l_{1}$ norm regression is employed to identify the points of the detected candidate target to avoid repeatedly detecting the same ship.

Step 3, the false alarm elimination. As illustrated in Figure 1, there are mainly two kinds of potential false alarms in GF-3 SAR images, named the bright lines, and the azimuth ambiguity. These false alarms may also occur in images generated from other SAR satellites, such as TerraSAR-X and COSMO-SkyMed [26,42]. The bright lines along the azimuth direction are caused by the ship sidelobes or the system noise [42], and the azimuth ambiguity is mainly formed due to aliasing [27]. The bright lines are removed via comparing the valid area of the candidate target with a pre-set value, and the azimuth ambiguity is eliminated by computing the displacements between the true target and its replicas.

Azimuth ambiguities frequently occur in GF-3 images with UFS mode, and the blue line in Figure 1 illustrates an example of azimuth ambiguity in a GF-3 image with UFS mode. This azimuth ambiguities elimination method cannot cope with the case when there is a true target located at the azimuth ghost position of another target. This issue can be addressed by using some complex features of the target, such as the contour or the context, however this will significantly increase the complexity of the algorithm. In addition, since this case occurs rarely, we still use the simple and efficient azimuth ambiguities elimination method.

### 2.2. Iterative CFAR Approach

The popular CFAR detector estimates the distribution of the sea clutter in a reference window which is moved over the image, and then determines the threshold that maintains a given false alarm rate for detection. There are two issues in the CFAR method, which are worth discussing. One is the selection for the statistical model of the sea clutter distribution, and the other is the selection of pixels used to estimate the distribution.

The gamma distribution, the K-distribution, and the mixture of gamma distributions are widely used for modeling the sea clutter distribution [15]. Although the sea clutter can be spiky and less homogeneous depending on the weather conditions and radar parameters, in which case K-distribution should be chosen, we use the homogeneous model, in part because this simplification can improve the efficiency of the algorithm and will not evidently deteriorate the result, in part because the adopted censoring scheme is only suitable for the homogeneous model. We select the gamma distribution as the model, since it is a good approximation for homogenous and multi-looked intensity images [11]. Let x be a random variable that follows the gamma distribution, and its probability density function (PDF) is defined as follows:

(1) $$p(x)={\left(\frac{a}{\mu }\right)}^{a}\frac{x^{a-1}e^{-xa/\mu }}{\Gamma (a)}$$

where a is the parameter controlling the shape of the distribution, μ is the mean value, and $\Gamma (a)=\int_{0}^{\infty }x^{a-1}e^{-x}dx$ is the gamma function. The cumulative distribution function (CDF) of x is defined as:

(2) $$P(x)=\frac{\gamma (a,xa/\mu )}{\Gamma (a)}$$

where $\gamma (a,b)=\int_{0}^{b}x^{a-1}e^{-x}dx$ is called as the lower incomplete gamma function. Given a set of sea clutter pixels ${\left\{x_{i}\right\}}_{i=1}^{n}$, μ and a can be obtained with the maximum likelihood estimator. The estimated $\hat{\mu }$ and $\hat{a}$ are computed according to Equations (3) and (4), and the detailed derivation can be found in [15]:

(3) $$\hat{\mu }=\frac{1}{n}\sum_{i=1}^{n}x_{i}$$

(4) $$\hat{a}=\frac{{\hat{\mu }}^{2}}{\frac{1}{n-1}\sum_{i=1}^{n}{\left(x_{i}-\hat{\mu }\right)}^{2}}$$

Once μ and a are determined, the decision threshold T under the estimated model can be computed via Equation (5), given a specified false alarm rate $P_{f}$:

(5) $$P_{f}=1-\frac{\gamma (\hat{a},T\hat{a}/\hat{\mu })}{\Gamma (\hat{a})}$$

By comparing the pixel value with T, the potential ship pixels can be detected. The traditional CFAR-based method usually selects a homogeneous area, and uses the pixels in that area for the estimation, although it is difficult to select such an area automatically. Recent studies in [16,17,18] indicate that the censoring scheme is effective for boosting the precision of the estimation by censoring out the potential target pixels. The iterative CFAR approach applies the censoring scheme, in which potential ship pixels are detected iteratively to refine the estimation step by step. The approach iteratively carries out the following steps until convergence: (1) estimate the sea clutter distribution using the sea clutter pixels; (2) implement the CFAR detection with the distributed model; (3) censor out both potential ship pixels and their eight-connected neighborhood pixels. At the beginning, all pixels are taken as the sea clutter pixels, that is to say, we do not employ an initial detection. It has been proved in [18] that censoring out the neighborhood pixels around potential ship pixels can further reduce the influence of such pixels when estimating the sea clutter distribution. It is noticed that the censoring scheme may neglect some sea clutter pixels, and the reduction of such amount of sea clutter pixels would not affect the accuracy of the estimation when a homogeneous model is used, however, if an heterogeneous model is used, the K-distribution model for example, those pixels should not be excluded. For the applied CFAR detection, the false alarm rate is set as 0.001%, and the size of the reference window is set as 600 m × 600 m. The iterative CFAR approach converges very fast, about five iterations in general. The iterative CFAR approach is summarized in Algorithm 1.
**Algorithm 1** The Iterative CFAR Approach**Input**: The input image.**Process**:(1)Take all pixels as the sea clutter pixels.(2)Repeat step (3) to (5) until convergence.(3)Estimate the sea clutter distribution using the sea clutter pixels.(4)Implement the CFAR detection with the distributed model. Compute the threshold T , and call every pixel with a value larger than T as the potential ship pixel.(5)Censor out both the potential ship pixels and their eight-connected neighborhood pixels.(6)Implement the CFAR detection with the final distributed model.**Output**: Potential ship pixels.

**Figure 3:**
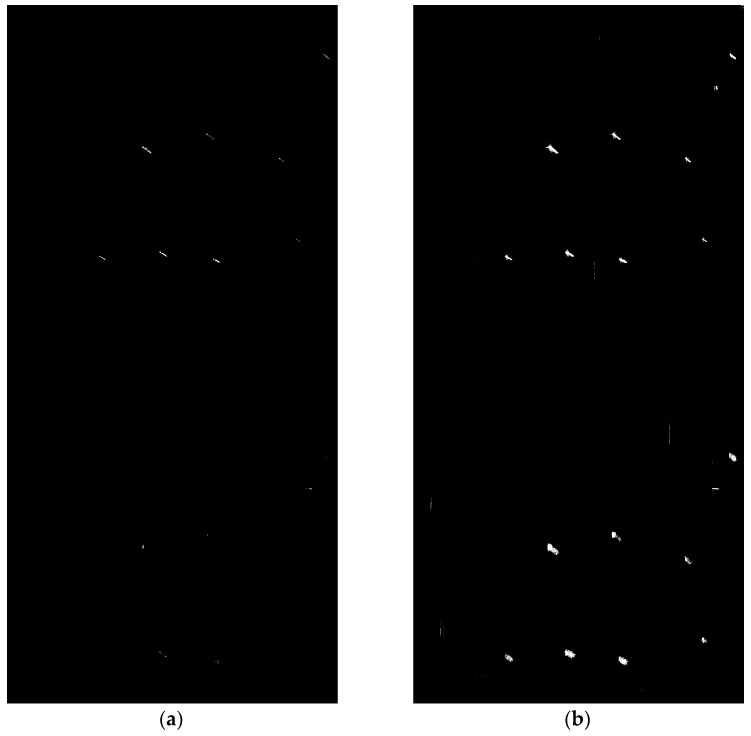
Detected results of the iterative CFAR approach after the first and the fourth iteration. (**a**) Detected result after the first iteration; (**b**) Detected result after the fourth iteration, the approach converges after four iterations, therefore it is also the final detected result of the iterative CFAR approach.

We apply the iterative CFAR approach on the SAR image in Figure 1, and show the detected result in Figure 3. Figure 3a,b correspond to the results after one and four iterations, respectively. As shown in Figure 3a, after the first iteration the detected result is already pretty good. However, an amount of ship pixels are not detected, which is likely to give rise to obstacles for the subsequent detection. Figure 3b gives the detected result after four iterations, and it is also the final result of the approach. It can be seen from Figure 3b that although almost all ship pixels have been detected, some false alarm points also appear in the detected result. These false alarm points can be removed after the false alarm elimination.

### 2.3. Mean-Shift Based Candidate Target Region Identification

**Figure 4:**
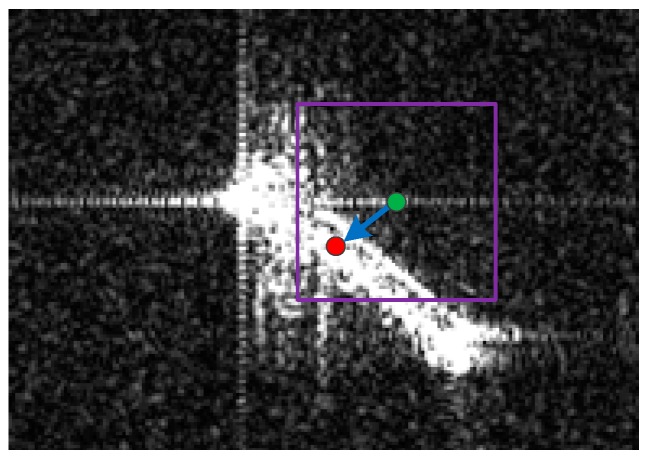
An illustration of the mean-shift based point selection.

In practice, it is necessary to outline the detected vessel as the output. There are three kinds of approaches for outlining the detected target, the connected domain or contour-based approach [22,43], the clustering-based approach [25], and the sliding window-based approach [44]. To efficiently identify the candidate target region, a mean-shift based method, which is similar with the approach in [44], is applied. In [44], the image is cut up into adjoining windows, and then the mean-shift operator is applied on each window, making it move towards the position where an object most likely locates in. Inspired by the object locating method in [44], we use the mean-shift operator to identify the position of candidate target region. The principle of the approach is illustrated in Figure 4, and the idea is quite straightforward. Given a potential ship pixel as the start point (the green point in Figure 4), we compute the weighted position of pixels in the (2r+1) × (2r+1) searching region around the start point which is shown using the purple box in Figure 4, and move the selected point to the computed position (the red point in Figure 4). It can be seen from the Figure 4 that after one mean-shift, the selected point is closer to the center point of the target. The mean-shift operation terminates until the position of the selected point converges, and the L×L region centered on the final selected point is taken as the candidate target region. The mean-shift operation makes the selected point move toward the position with locally densest target pixels, consequently can be used for locating the candidate target. To avoid repeatedly detecting the same ship, we introduce a selection matrix that records which pixels have been identified as points of the detected candidate targets, and these pixels are no longer taken as either the start or the final selected point for the mean-shift operation. For a candidate target region, these pixels are referred to as valid points of the candidate target and can be obtained via the $l_{1}$ norm regression which will be detailed later.

After giving the intuitive explanation, we present the method in a formal way. The symbols used in the proposed method are introduced firstly for clarity. Let f denote the image to be processed, and its element $f_{xy}$ denotes the intensity of the pixel with position (x,y). Let g denote the binary result of the iterative CFAR approach, with pixel value of one indicating a potential ship pixel and zero indicating a sea clutter pixel. Denote p as the selection matrix with elements $p_{xy}$, and $p_{xy}=1$ indicates that the corresponding pixel can be taken as a selected point. p is initialized as g, that is to say, at the beginning all potential ship pixels can be selected. It is assumed that a brighter point is more likely to be a real ship pixel, therefore we sort the potential ship pixels in descending order to make sure a brighter point will be taken earlier. Take the sorted potential ship pixels one by one, and check the selection matrix to see if the pixel can be taken as a selected point. When it can be, select it as the start point and apply the mean-shift operation until convergence. Suppose the start point locates in $\left(x_{0},y_{0}\right)$, and after the tth iteration, it locates in $\left(x_{t},y_{t}\right)$, then the position of the selected point after the (t+1)th iteration can be calculated as follows:

(6) $$\begin{array}{l} x_{t+1}=\sum_{(x,y)\in N_{t}}xf_{xy}/\sum_{(x,y)\in N_{t}}f_{xy}\\ y_{t+1}=\sum_{(x,y)\in N_{t}}yf_{xy}/\sum_{(x,y)\in N_{t}}f_{xy} \end{array}$$

where $N_{t}$ is the set containing location of pixels used to estimate the updated position of the selected point, which can be formulated as follows:

(7) $$N_{t}=\{(x,y)|x\in [x_{t}-r,x_{t}+r],y\in [y_{t}-r,y_{t}+r],g(x,y)=1\}$$

where, r is the radius of the searching region. Equation (6) computes the weighted position of the potential ship pixels in the searching region, and essentially it is a mean-shift operation. The mean-shift process terminates when the position of the selected point does not change. Check the selection matrix to see if the final selected point can be taken, and when it can be, the L×L region centering on the final selected point is detected as a candidate target region.

**Figure 5:**
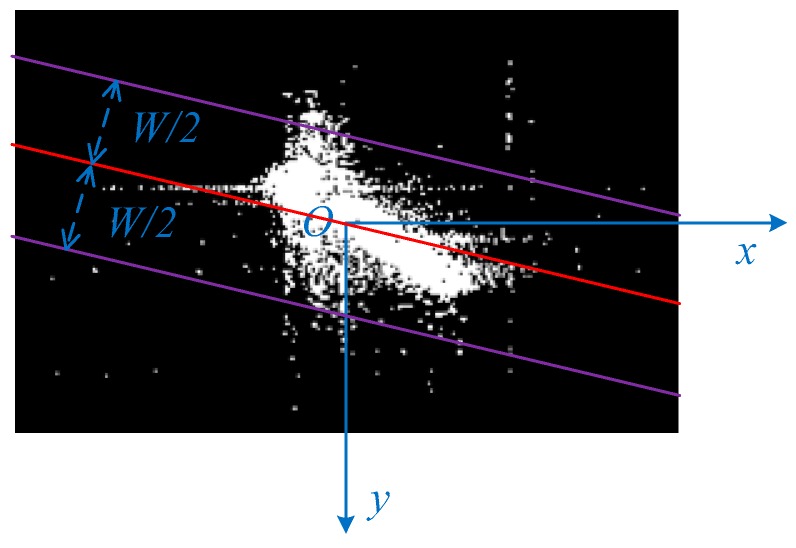
The principal axis extracted through the $l_{1}$ norm regression.

To avoid repeatedly detecting the same ship, the selection matrix should be updated when a candidate target region is detected. The potential ship pixels belonging to the candidate target are called valid points of the target, and we update the selection matrix by setting the pixels corresponding to the valid points in p as zero, so that these valid points would not be taken as either the start or the final selected point. Taking all potential ship pixels in the L×L region as the valid points is not quite reasonable, in part because some potential ship pixels may belong to other ships, in part because ship is a strip shaped object and the ship pixels are usually concentrated along one direction. To detect the valid points of the candidate target, we apply the $l_{1}$ norm regression to extract the principal axis of the candidate target and the potential ship pixels with distance to the principal axis less than W/2 are identified as the valid points, where W is the maximum width of the ship to be detected. The principal axis of the candidate target can be obtained by estimating the principal direction of the potential ship pixels in the L×L candidate target region. Principal component analysis (PCA) or the $l_{2}$ norm regression [45] can be used to estimate the principal direction, however both of them are highly sensitive to outliers, which are common in SAR images due to the sidelobe and the system noise. An effective method to estimate parameters in a regression model that is insensitive to outliers is the $l_{1}$ norm regression [40]. We establish the coordinate system so that the origin point locates in the center of the candidate target region, as illustrated in Figure 5. The problem can be expressed as follows, given a set of data ${\left\{(x_{i},y_{i})\right\}}_{i=1}^{n}$, estimate the parameter w so that the line y=wx can fit the data with the least absolute deviations. Let $x={\left[x_{1},\ldots ,x_{n}\right]}^{T}$ and $y={\left[y_{1},\ldots ,y_{n}\right]}^{T}$, the problem can be formulated as follows:

(8) $$\underset{w}{\min}\frac{1}{2}{\left\|y-wx\right\|}_{1}$$

The above problem can be solved efficiently in an iterative manner. Suppose $w_{k}$ represents the estimated parameter after the kth iteration. Given $w_{k-1}$, $w_{k}$ can be obtained by expanding the objective function and replacing it with an approximating representation:

(9) $$\frac{1}{2}{\left\|y-w_{k}x\right\|}_{1}=\frac{1}{2}\sum_{i=1}^{n}\frac{{\left(y_{i}-w_{k}x_{i}\right)}^{2}}{\left|y_{i}-w_{k}x_{i}\right|}\approx \frac{1}{2}\sum_{i=1}^{n}\frac{{\left(y_{i}-w_{k}x_{i}\right)}^{2}}{\left|y_{i}-w_{k-1}x_{i}\right|}=J(w_{k})$$

The solution of $\min_{w_{k}}J(w_{k})$ can be simply obtained by letting $J'(w_{k})=0$, which can be written analytically as follows:

(10) $$w_{k}=\frac{\sum_{i=1}^{n}x_{i}y_{i}/\rho_{i}^{k-1}}{\sum_{i=1}^{n}x_{i}^{2}/\rho_{i}^{k-1}}$$

where $\rho_{i}^{k-1}=\left|y_{i}-w_{k-1}x_{i}\right|$, and in practice we set $\rho_{i}^{k-1}$ as $\left|y_{i}-w_{k-1}x_{i}\right|+\varepsilon$ to assure the numerical stability of the method, where ε is a small positive number and set as 0.01 in the experiment. The initial estimation $w_{0}$ is computed by solving the following $l_{2}$ norm regression problem. Although the $l_{2}$ norm regression is sensitive to outliers, it can be applied to get a good initial solution:

(11) $$w_{0}=\arg\min_{w}\frac{1}{2}{\left\|y-wx\right\|}_{2}$$

By letting the derivative of the objective function in (11) with respective to w equal zero, $w_{0}$ can be computed analytically as:

(12) $$w_{0}=\frac{\sum_{i=1}^{n}x_{i}y_{i}}{\sum_{i=1}^{n}x_{i}^{2}}$$

Figure 5 shows an example of the principal axis extraction and the valid point identification, in which the red line indicates the extracted principal axis using the $l_{1}$ norm regression, and the two purple lines show the boundaries of the valid point.

There are three parameters to be decided in the mean-shift based candidate target region identification approach, that is the radius of the searching region r, the side length of the candidate target region L and the maximum width of the ship to be detected W. By setting r as different values, we find that in a wide range, r only affects the convergence speed, and has almost no effect on the position of the final selected point. We set r as 50 m in the experiment, and under that setting the mean-shift process converges extremely fast, about two to four iterations in general. The candidate target region is supposed to contain the whole target, therefore L is set as 300 m, which is the maximum length of the vessel to be detected. It is supposed that most ship pixels can be included between the two boundaries, thus W is set as 80 m, which is the maximum width of the vessel to be detected. The mean-shift based candidate target region identification is summarized in Algorithm 2.
**Algorithm 2** Mean-shift Based Candidate Target Region Identification**Input**: The input image f and the binary result of the iterative CFAR approach g.**Process**:(1)Initialize the selection matrix p as g. Set the size of the selected region L×L, the radius of the searching region r, the side length of the candidate target region L and the maximum width of the ship to be detected W.(2)Take out the potential ship pixels whose values are one in g, and sort them according to their intensities in descending order.(3)Take the potential ship pixels in sequence. For each potential ship pixel, check p to see if the pixel can be taken as a selected point. When it can be, select it as a start point and move to the next step, otherwise repeat step (3) to take the next potential ship pixel.(4)Do the mean-shift operation until convergence.(5)For the final selected point, Check p to see if it can be taken as a selected point. When it can be, go to step (6), otherwise go to step (3) to take the next potential ship pixel.(6)The L×L region centering on the final selected point is detected as a candidate target region. Employ the $l_{1}$ norm regression to extract the principal axis of the target, and identify the valid points of the target. Update the selection matrix p by setting the corresponding pixels of valid points in p as zero, so that the valid points of the target are no longer taken as either the start or the final selected point.(7)Go to step (3) to take the next potential ship pixel.**Output**: Candidate target regions.

### 2.4. False Alarms Elimination

False alarms are eliminated with a two-step technique. In the first step, bright lines are removed with the help of the valid area. For a candidate target region, we define its valid area as the multiplication of the pixel widths along the range and the azimuth directions and the number of the valid points. The bright line can be removed by comparing the valid area of the candidate target with a pre-defined threshold $T_{f}$, since the difference of the valid area between the true target and the bright line is large. In general, the valid area of the true target is more than 1000 $m^{2}$, while the bright line has valid areas less than 200 $m^{2}$, therefore we set $T_{f}$ as 1000 $m^{2}$ to distinguish the true target from the bright line.

In the second step, azimuth ambiguities are excluded by computing the relative distances between the target and its replicas. The displacement between a true target and its ith order azimuth ambiguity is as follows [29]:

(13) $$d_{i}=i\frac{\lambda R}{2V}f_{p},i=\pm 1,\pm 2,\ldots$$

where λ is the wavelength, R is the range distance between the target and the satellite, V is the velocity of the satellite, and $f_{p}$ is the PRF of the single channel. As done in [3,26,30], we only take the first order azimuth ambiguity into consideration, since in general the higher order azimuth ambiguities would not form false alarms after the CFAR detection. The first order azimuth ambiguity can be eliminated using the following technique. Firstly, sort the candidate targets according to their mean intensities in descending order, so that the true target will be detected before its replicas are taken out. Secondly, check the candidate targets in sequence. If there is a detected true target above or below it along the azimuth direction with the distance $d_{1}$, recognize it as an azimuth ambiguity and remove it, otherwise, recognize it as a true target.

## 3. Results and Discussion

### 3.1. Experimental Data

Three GF-3 SAR images with UFS mode are used as the experimental data, and the detailed information of the three images is listed in Table 1. The DH polarization representing the signal is transmitted once with the horizontal polarization, and received twice with the horizontal and the vertical polarizations, respectively. SLP is short for single look power image, and it is one product type of the GF-3 satellite. These images are single-looks, and have not been radiometrically calibrated. We do the multi-look processing to suppress the speckle noise, and the number of looks is 2 in both range and azimuth directions. Rng and Az in Table 1 are short for the range and the azimuth directions respectively, and the resolution, the size, and the pixel width are all referred to the values before performing the multi-look processing.

**Figure 6:**
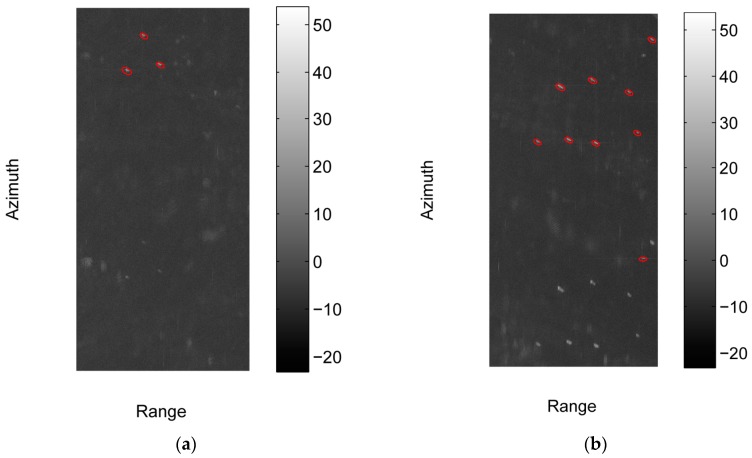

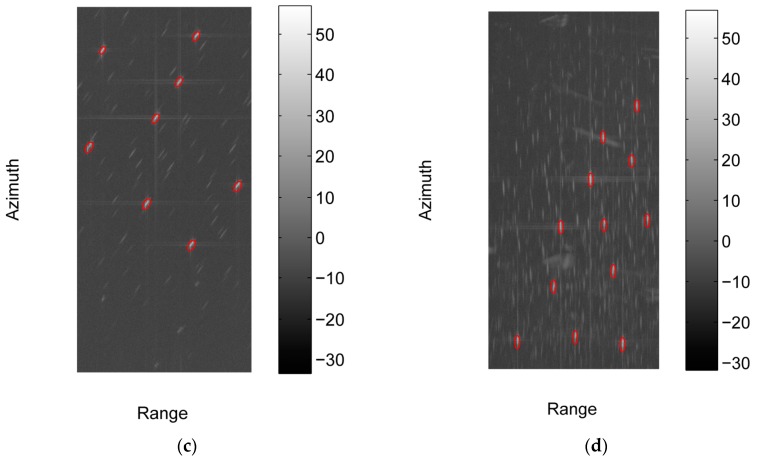
The four sub-images used in the experiment and the manually annotated ships in them. The intensities of these images in dB are shown in the figure. (**a**) The intensity of sub-image #1 in dB; (**b**) The intensity of sub-image #2 in dB; (**c**) The intensity of sub-image #3 in dB; (**d**) The intensity of sub-image #4 in dB.

We crop four sub-images with the size of 3260 × 6879 [Rng × Az] (pixels) from the three images, where sub-image #1 and #2 are cropped from image #1, and sub-image #3 and #4 are cropped from image #2 and #3 respectively. Figure 6 shows the intensities of the four sub-images in dB. We do not acquire the automatic identification system (AIS) data of the corresponding region. To assess the detection performance, the ground truth is obtained via visually inspection by professional SAR interpreters. The manually annotated ships are marked with red circles in Figure 6.

### 3.2. Detected Results of the Proposed Method

**Figure 7:**
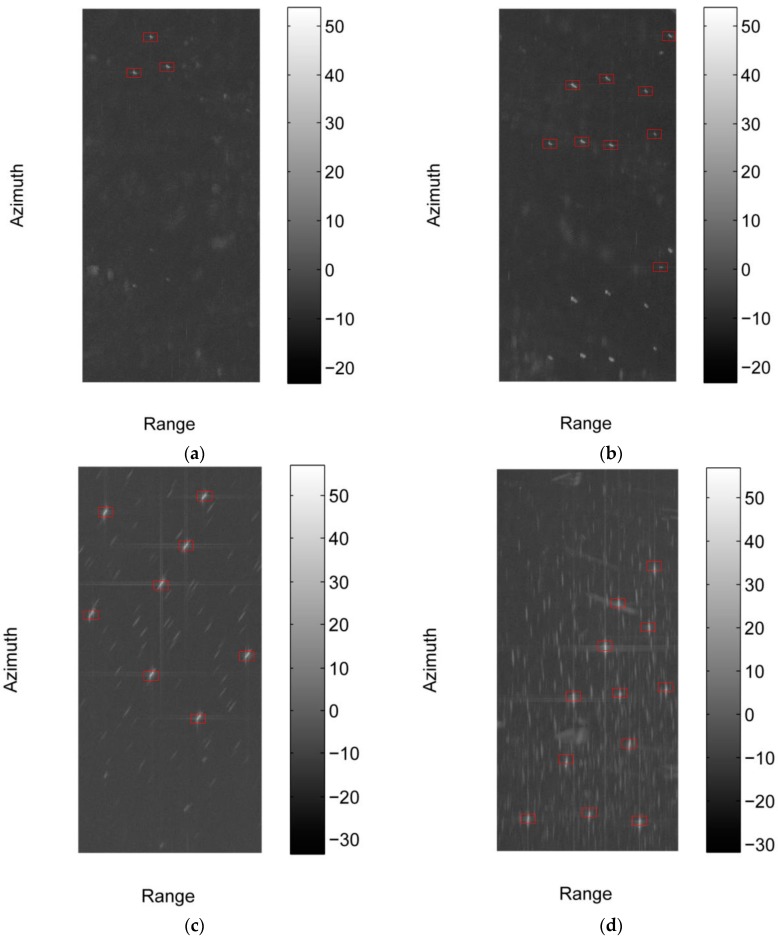
Detected results of the proposed method on sub-image #1 to #4, and the intensities of these images in dB are shown in the figure. (**a**) Detected result of sub-image #1; (**b**) Detected result of sub-image #2 (**c**) Detected result of sub-image #3; (**d**) Detected result of sub-image #4.

We implement the proposed method with C++ programming and carry out the experiment on a computer with Inter Core at 3.2 GHz. The intensities of the four sub-images and the detected results of the proposed method are shown in Figure 7, and the red box in the image indicates the detected vessel. Sub-image #1 and #2 contain some bright lines and azimuth ambiguities, thus are suitable for testing the false alarms elimination ability of the proposed method. The backgrounds of sub-image #1 and #2 are relatively simple. To fully validate the effectiveness of the proposed method, we select sub-image #3 and #4, in which a lot of azimuth ambiguities exist. As shown in Figure 7a–d, the detected results of the proposed method are in accordance with the manually annotated results, that validates the effectiveness of the proposed method. The cost times of the proposed method on the four sub-images with the size of 3260 × 6879 pixels are 3.759, 3.354, 3.510, and 3.525 s respectively, which makes the proposed method practical for real applications.

### 3.3. Comparison with State-of-the-Art

In this section, we compare our method with three state-of-the-art SAR ship detection methods, including TS-SEG-CFAR in [15], IICS in [18], and SLIC-CFAR in [25]. All three methods focus on the potential ship pixels detection, and in their work detected results are presented using the binary indication image, in which pixels with value of one are the ship points and zero are the sea clutter points. We just show the binary indication image to present the detected result of the three methods. Our method does not generate such binary indication image, instead detected boxes are plotted in the input image to show the positions of the detected targets.

**Figure 8:**
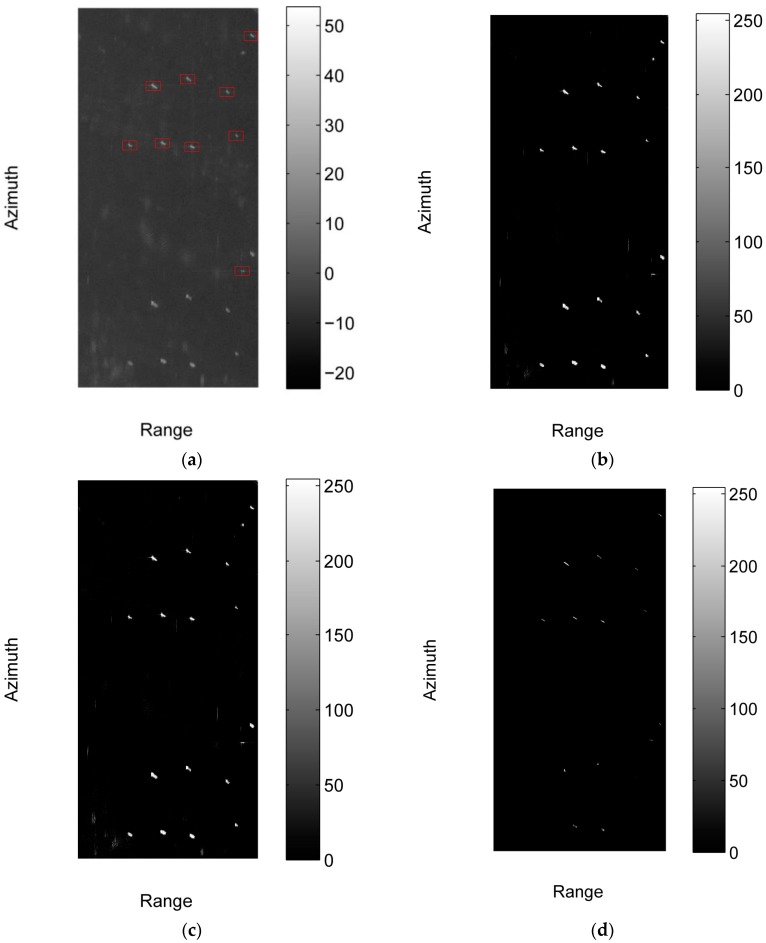
Comparison of detected results of various methods on sub-image #2. (**a**) Detected result of the proposed method, and the intensity of the image in dB is shown in this figure; (**b**) Detected result of the TS-SEG-CFAR method; (**c**) Detected result of the IICS method; (**d**) Detected result of the SLIC-CFAR method.

**Figure 9:**
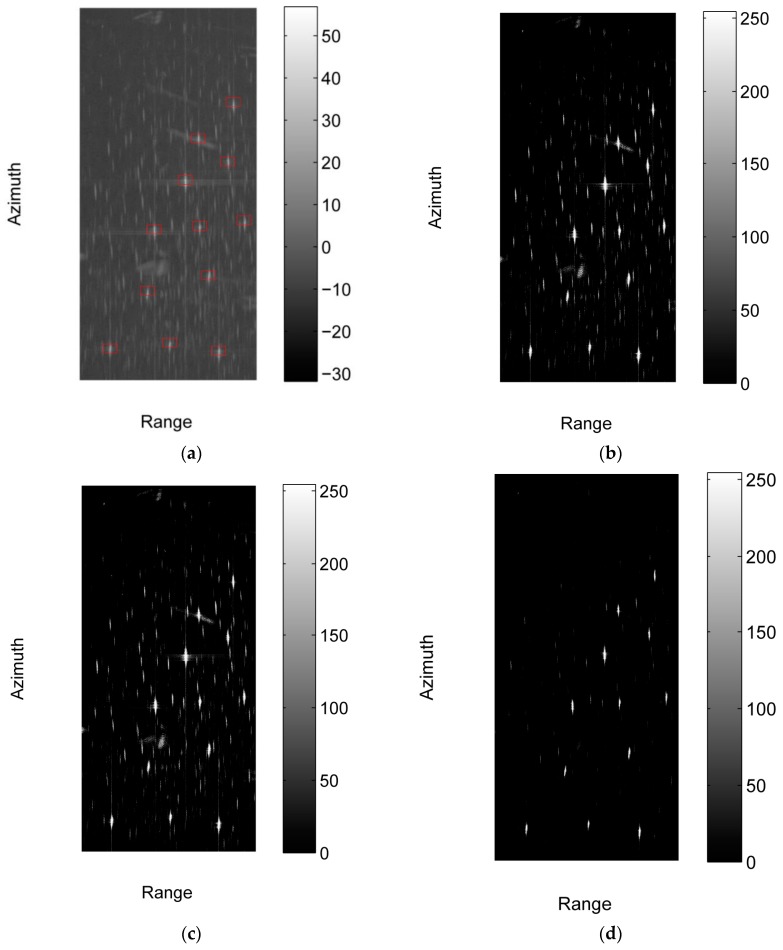
Comparison of detected results of various methods on sub-image #4. (**a**) Detected result of the proposed method, and the intensity of the image in dB is shown in this figure; (**b**) Detected result of the TS-SEG-CFAR method; (**c**) Detected result of the IICS method; (**d**) Detected result of the SLIC-CFAR method.

Figure 8 compares the detection results of the various methods on sub-image #2. It can be imagined that by combining some post-processing, such as the clustering algorithm, TS-SEG-CFAR, IICS, and SLIC-CFAR can detect the true targets, however this will generate some false targets in the results, which are not taken into account in these methods. Figure 9 compares detected results of various methods on sub-image #4. There are many false alarm points in the results of TS-SEG-CFAR and IICS. In contrast with TS-SEG-CFAR and IICS, SLIC-CFAR detects much less false alarm points, however at the expense of much more running time (see Table 2).

It is worth pointing out that the main difference between the detectors is the capability to reject false alarms by using the false alarm elimination approach in the third stage. If such approach were to be applied to the other detectors, then the performance would be very similar, since the probability of detection for these detectors on this type of ships are comparable. We prefer our proposed detection stage to the others we compared it with because it is much faster. The cost times of different methods on the four sub-images are listed in Table 2, and it is clear that compared with the other three methods the proposed method runs orders of magnitude faster. The main time-consuming part of the TS-SEG-CFAR method is the estimation of the sea clutter distribution, a more complex model, the mixture of gamma distributions model, is used. The expectation maximization (EM) algorithm is applied to estimate the parameters, which is time-consuming. What’s more, since the class number of the mixture model is not known, the method has to use mixture models with different class numbers and apply Pearson’s $\chi^{2}$ test to determine an optimal class number [15]. In contrast with TS-SEG-CFAR, we select a simpler model, the gamma distribution model. Although this simpler model is not as accurate as the mixture of gamma distributions model in terms of representing the sea clutter in the heterogeneous region, the efficiency of the algorithm is increased evidently due to this simplification. Compared with the IICS method [18], the proposed method omits the initial detection step which is slightly time-consuming, consequently has higher efficiency. The SLIC-CFAR method [25] takes the longest running time among the compared methods, mainly because SLIC is a segmentation algorithm that divides the image into many superpixels, which requires much more running time. Different from SLIC-CFAR, which uses superpixel to get the candidate target region, the proposed method applies the mean-shift operation on each potential ship pixel to get the region, which is much more efficient.

## 4. Conclusions

In this paper, we propose a fast vessel detection method in GF-3 SAR images with UFS mode. The method consists of three parts: the iterative CFAR-based potential ship pixel detection, the mean-shift-based candidate target region identification and the false alarm elimination. The iterative CFAR approach iteratively censors out the potential ship pixels to reduce the influence of such pixels on the estimation of the sea clutter distribution. The mean-shift operation is applied on each potential ship pixel to make it move towards the position with locally densest target pixels. The L×L region centered on the final selected point of mean-shift is identified as the candidate target region. To avoid repeatedly detecting the same ship, the $l_{1}$ norm regression is employed to extract the principal axis of the candidate target, and the pixel with distance to the principal axis less than a pre-defined value is recognized as the pixel of the candidate target. Such pixel is referred to as the valid point of the target and is no longer taken as the selected point for the mean-shift operation. Finally false alarms are excluded from the candidate targets to refine the detected result. By analyzing the vessel characteristic in GF-3 SAR images with UFS mode, we apply a two-step false alarm elimination approach for removing two kinds of potential false alarms, namely bright lines and azimuth ambiguity. We define the valid area of a candidate target as the multiplication of pixel widths along the range and the azimuth directions and the number of the valid point. The bright lines are removed by comparing the valid area of the candidate target with a pre-defined threshold, since the difference of the valid area between the true target and the bright line is large. The azimuth ambiguity is eliminated by computing the displacement between the true target and its first order azimuth ambiguity. Experimental results on four sub-images cropped from three GF-3 SAR images with UFS mode demonstrate the effectiveness of the proposed method. False alarms can be effectively excluded by the proposed method, even when the background is complex. Comparison results with three state-of-the-art methods validate that the proposed method is competitive in terms of the detected result. More importantly, the proposed method runs orders of magnitude faster than the three compared methods, which makes our method more practical for real applications.

## Figures and Tables

**Table 1:** The detailed information of the three GF-3 SAR images used in the experiment.

Parameter	Image #1	Image #2	Image #3
Region	East China sea	Bohai sea	Bohai sea
Imaging mode	UFS	UFS	UFS
Nominal resolution (m)	3	3	3
Resolution [Rng × Az] (m)	3.3 × 2.4	3.3 × 2.4	3.3 × 2.4
Number of looks [Rng × Az]	1 × 1	1 × 1	1 × 1
Wave length (m)	0.0555	0.0555	0.0555
Satellite direction	Descending	Descending	Descending
Look direction	Right	Right	Right
Polarization	DH	DH	DH
Product level	1	1	1
Product type	SLP	SLP	SLP
Incidence angles (degree)	43.75–45.29	48.53–49.79	48.51–49.80
Size [Rng × Az] (pixel)	20316 × 20207	19,443 × 21,643	19,905 × 21,640
Pixel width [Rng × Az] (m)	1.124 × 1.794	1.124 × 1.705	1.124 × 1.705

**Table 2:** The cost times (s) of different methods on the four sub-images.

Method	Sub-Image #1	Sub-Image #2	Sub-Image #3	Sub-Image #4
TS-SEG-CFAR	100.783	102.096	101.261	101.646
IICS	43.683	43.972	43.873	43.795
SLIC-CFAR	1091.410	1094.040	1095.644	1094.261
Our method	3.759	3.354	3.510	3.525
